# Intratumoural evolutionary landscape of high-risk prostate cancer: the PROGENY study of genomic and immune parameters

**DOI:** 10.1093/annonc/mdx355

**Published:** 2017-07-19

**Authors:** M. Linch, G. Goh, C. Hiley, Y. Shanmugabavan, N. McGranahan, A. Rowan, Y. N. S. Wong, H. King, A. Furness, A. Freeman, J. Linares, A. Akarca, J. Herrero, R. Rosenthal, N. Harder, G. Schmidt, G. A. Wilson, N. J. Birkbak, R. Mitter, S. Dentro, P. Cathcart, M. Arya, E. Johnston, R. Scott, M. Hung, M. Emberton, G. Attard, Z. Szallasi, S. Punwani, S. A. Quezada, T. Marafioti, M. Gerlinger, H. U. Ahmed, C. Swanton

**Affiliations:** 1 Translational Cancer Therapeutics Laboratory, UCL Cancer Institute, London, UK;; 2 Department of Medical Oncology, University College London Hospitals NHS Foundation Trust , London, UK;; 3 Bill Lyons Informatics Centre, UCL Cancer Institute, London, UK;; 4 Division of Cancer Studies, King's College London, London, UK;; 5 Translational Cancer Therapeutics Laboratory, The Francis Crick Institute, London, UK;; 6 Division of Surgery and Interventional Science, University College London, London, UK;; 7 Cancer Immunology Unit, UCL Cancer Institute, London, UK;; 8 Research Department of Haematology, UCL Cancer Institute, London, UK;; 9 Department of Histopathology, University College London Hospitals NHS Foundation Trust, London, UK;; 10 Definiens AG, Munich, Germany;; 11 Department of Bioinformatics and Biostatistics, The Francis Crick Institute, London, UK;; 12 Cancer Genomics Laboratory, The Francis Crick Institute, London, UK;; 13 Experimental Cancer Genetics, Wellcome Trust Sanger Institute, Cambridge, UK;; 14 The Urology Centre, Guy's and St. Thomas' NHS Foundation Trust, London, UK;; 15 Department of Urology, UCLH NHS Foundation Trust, London, UK;; 16 Centre for Medical Imaging, Universtiy College London, London, UK;; 17 Centre for Evolution and Cancer, The Institute of Cancer Research, London, UK;; 18 Department of Medical Oncology, Royal Marsden Hospital, London, UK;; 19 Centre for Biological Sequence Analysis, Technical University of Denmark, Lyngby, Denmark;; 20 Computational Health Informatics Program (CHIP), Harvard Medical School, Boston, USA;; 21 MTA-SE-NAP Brain Metastasis Research Group, Semmelweis University, Budapest, Hungary;; 22 Division of Surgery, Department of Surgery and Cancer, Imperial College London, UK;; 23 Department of Urology, Imperial College Healthcare NHS Trust, London, UK

**Keywords:** prostate cancer, intratumoural heterogeneity, neoepitopes, tumour infiltrating lymphocytes, wnt signalling, mismatch repair

## Abstract

**Background:**

Intratumoural heterogeneity (ITH) is well recognised in prostate cancer (PC), but its role in high-risk disease is uncertain. A prospective, single-arm, translational study using targeted multiregion prostate biopsies was carried out to study genomic and T-cell ITH in clinically high-risk PC aiming to identify drivers and potential therapeutic strategies.

**Patients and methods:**

Forty-nine men with elevated prostate-specific antigen and multiparametric-magnetic resonance imaging detected PC underwent image-guided multiregion transperineal biopsy. Seventy-nine tumour regions from 25 patients with PC underwent sequencing, analysis of mutations, copy number and neoepitopes combined with tumour infiltrating T-cell subset quantification.

**Results:**

We demonstrated extensive somatic nucleotide variation and somatic copy number alteration heterogeneity in high-risk PC. Overall, the mutational burden was low (0.93/Megabase), but two patients had hypermutation, with loss of mismatch repair (MMR) proteins, MSH2 and MSH6. Somatic copy number alteration burden was higher in patients with metastatic hormone-naive PC (mHNPC) than in those with high-risk localised PC (hrlPC), independent of Gleason grade. Mutations were rarely ubiquitous and mutational frequencies were similar for mHNPC and hrlPC patients. Enrichment of focal 3q26.2 and 3q21.3, regions containing putative metastasis drivers, was seen in mHNPC patients. We found evidence of parallel evolution with three separate clones containing activating mutations of β-catenin in a single patient. We demonstrated extensive intratumoural and intertumoural T-cell heterogeneity and high inflammatory infiltrate in the MMR-deficient (MMRD) patients and the patient with parallel evolution of β-catenin. Analysis of all patients with activating Wnt/β-catenin mutations demonstrated a low CD8+/FOXP3+ ratio, a potential surrogate marker of immune evasion.

**Conclusions:**

The PROGENY (PROstate cancer GENomic heterogeneitY) study provides a diagnostic platform suitable for studying tumour ITH. Genetic aberrations in clinically high-risk PC are associated with altered patterns of immune infiltrate in tumours. Activating mutations of Wnt/β-catenin signalling pathway or MMRD could be considered as potential biomarkers for immunomodulation therapies.

**Clinical Trials.gov Identifier:**

NCT02022371

## Introduction

Prostate cancer (PC) is the second most common malignancy in men with an incidence of 1.1 million men per year leading to an estimated 307,000 deaths worldwide [1]. While the prognosis of clinically low-risk PC is excellent [2], there is significant mortality associated with clinically high-risk disease, with approximately 20%–25% 10-year cancer-specific mortality despite radical treatments [3].

A key challenge in PC is to identify patients with potentially lethal disease while avoiding the morbidity of overtreatment in patients with indolent disease. Accurate risk stratification has been confounded by underestimation of disease burden using standard transrectal ultrasound-guided biopsies and extensive tumour heterogeneity of primary PC [4–6]. A recent improvement on transrectal ultrasound-guided biopsies is targeted magnetic resonance imaging (MRI)-guided biopsies that increase the likelihood of sampling clinically significant disease [7].

Multi-regional sampling of tumours allows measurement of intratumoural heterogeneity, a prognostic entity in PC [8]. To date, in-depth exploration of genomic ITH with multiregion sequencing (M-Seq) in the primary PC has relied on prostatectomy patient series [9, 10] to provide good-quality tissue; however, this has enriched for clinically low- and intermediate-risk disease [5, 6].

Tumour infiltrating lymphocyte density has been shown, albeit inconsistently, to be prognostic in PC [11–13]. The impact of tumour genetics on prostate immunobiology is unclear and deciphering this could improve risk stratification, prognostication and immunotherapeutic approaches.

We conducted the PROGENY study (PROstate cancer GENomic heterogeneitY) to attain high-quality multi-regional prostate biopsies to determine the driver and evolutionary events of clinically high-risk PC at the time of diagnosis and to correlate genomic and immune parameters.

## Methods and materials

### Patient selection

Between September 2013 and December 2015, 49 men with a prostate-specific antigen ≥15, a multi-parametric MRI detectable lesion in the prostate and no prior prostate-directed biopsies or treatments were enrolled into the PROGENY study, with local ethics committee approval. Of these, 23 patients and a further 2 contemporaneous patients from the institutional biobank met the criteria for the planned genetic and T-cell analysis (supplementary Table S1 and Figure S1, available at *Annals of Oncology* online).

### Tissue procurement

Multi-regional PC biopsies were obtained using multi-parametric MRI, image-fusion transperineal template targeting as described previously [7] (supplementary Figure S2, available at *Annals of Oncology* online). Blood samples were obtained before the biopsy for isolation of germline DNA.

### Sequencing studies

Tumour DNA was extracted using the Allprep Micro Kit (Qiagen, CA) and germline DNA extracted with the DNeasy Blood & Tissue Kit (Qiagen, MD) following the manufacturer’s instructions. Further details are contained in the supplementary data, available at *Annals of Oncology* online.

### Immunohistochemistry

Single and multiplexed IHC was carried out as described previously [14]. Antibody details are in supplementary Table S2, available at *Annals of Oncology* online. TM and ML jointly carried out quantification of inflammatory infiltrate (INIF), blinded to patient characteristics. Samples with ≥8% (median of all samples) INIF (15/25 patients) in any one region were subjected to digital image analysis. These correlated well with the manual estimation (*R*^2^ = 0.71) (supplementary Figures S3 and S4, available at *Annals of Oncology* online).

## Results

### The extent of intratumoural heterogeneity in high-risk PC

**Figure 1.**
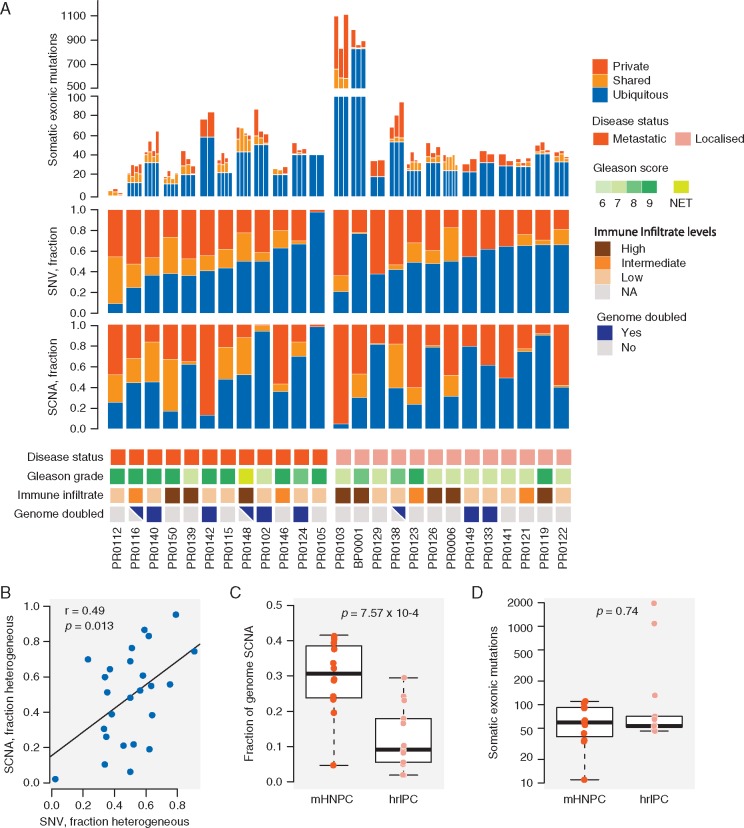
Intratumoural heterogeneity in prostate cancer at the somatic nucleotide variation (SNV) and somatic copy number alteration (SCNA) levels. (A) Number of somatic exonic mutations identified in each tumour region, fraction of SNVs and SCNAs that were ubiquitous (present in every tumour region of a given patient) (blue), shared (present in more than one tumour region, but not all) (light orange) or private (present in only one tumour region) (dark orange). Data tracks below indicate if patient was metastatic on presentation (red), Gleason grade (shades of green), level of tumoural inflammatory infiltrate (shades of brown), and if the tumour had undergone whole-genome doubling (purple, triangle indicating heterogeneous genome doubling). (B) Scatterplot showing correlation between degree of SNV and SCNA heterogeneity. (C and D) Boxplots comparing the fraction of genome affected by SCNA and SNV mutational burden in metastatic hormone naive prostate cancer (mHNPC) versus high-risk localised prostate cancer (hrlPC).

Across 25 prospectively recruited patients, M-Seq from 79 tumour regions identified a total of 4484 exonic somatic nucleotide variations (SNV) (3382 non-silent), of which 1962 were ubiquitous, 495 were shared and 2027 were private (Figure 1A). The overall estimation of exonic SNV burden was 0.93 mutations per megabase (median, range, 0.18–33 per megabase), consistent with prior studies in PC [15].

The overall fraction of the genome subject to somatic copy number alterations (SCNAs) was 23.1% (median, range 1.9%–41.6%). Of this fraction, a median of 52.3% (range 2.1%–95.3%) was heterogeneous (Figure 1A). The degree of SNV and SCNA heterogeneity among the tumours was positively correlated (Figure 1B) (*r* = 0.49, *P *= 0.013, Pearson’s).

Two patients, BP0001 and PR0103, had markedly elevated SNV rates. BP0001 had a previous diagnosis of Lynch Syndrome and was found to harbour a germline mutation in *MSH6* (p.G39E, rs1042821) and a somatic heterozygous deletion encompassing the region encoding for *MSH2* and *MSH6*, resulting in a hemizygous variant in *MSH6*. PR0103 had a somatic 10 Mb deletion overlapping *MSH2* and *MSH6* and a 5 kb somatic deletion across *MSH2*, leading to biallelic loss of *MSH2*. IHC of MSH2 and MSH6 in both of these patients showed complete loss of protein expression in the tumours (supplementary Figures S5 and S6, available at *Annals of Oncology* online).

### Genomic events enriched in patients presenting with metastatic disease

After the diagnostic biopsy, 12/25 patients were found to have metastatic disease on imaging (mHNPC) and 13 patients had localised PC with high risk for metastatic disease (hrlPC). mHNPC primary tumours had significantly higher burden of SCNAs compared with hrlPC tumours (29.6% ± 10.6% versus 12.5% ± 8.9%, *P *= 7.57 × 10^−4^, Mann–Whitney *U* test) (Figure 1C) and this was independent of Gleason grade. Comparing mHNPC and hrlPC patients, there was no significant difference in the proportion of heterogeneous SCNAs (*P* = 0.89, Mann–Whitney *U* test), overall mutational burden (*P *= 0.74, Mann–Whitney *U* test ) (Figure 1D), or proportion of heterogeneous mutations (*P* = 0.11, Mann–Whitney *U* test).

**Figure 2.**
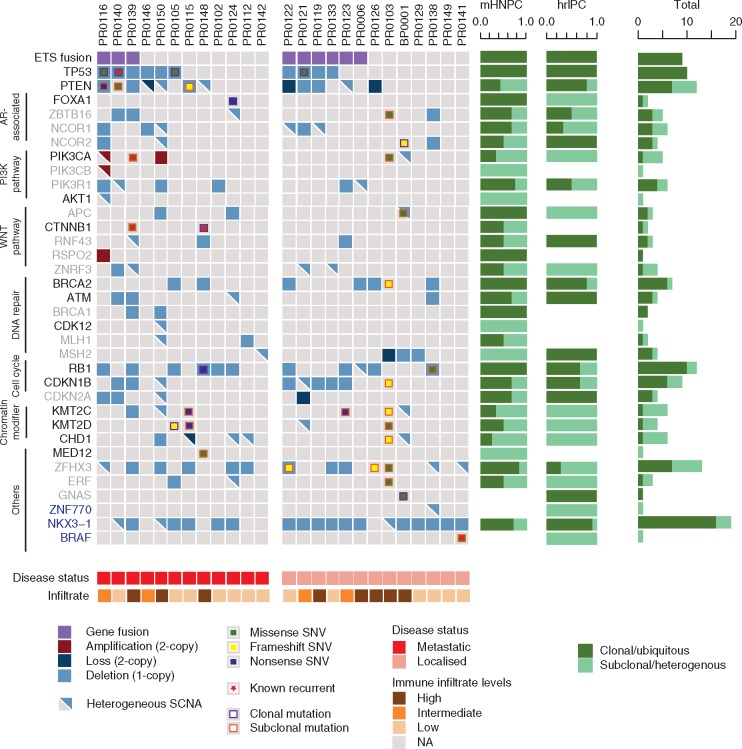
Clonal and subclonal driver events in prostate cancer. List of driver genes previously reported as significantly mutated in primary prostate cancer (blue), metastatic castrate-resistant prostate cancer (mCRPC) (grey), or both (black). Ubiquitous ETS fusion (purple), homozygous loss (dark blue), heterozygous deletion (blue), amplification (red) in each tumour is depicted by a coloured square, and heterozygous events are indicated with a triangle. Nonsynonymous mutations are depicted as smaller squares, whether missense (green), frameshift (yellow) or nonsense (dark blue). Clonal and subclonal mutations are indicated by a purple and orange outline, respectively. Known recurrent mutations in *TP53*, *PIK3CA, CTNNB1* and *BRAF* are indicated with a red star. The barplots on the right are an aggregate of clonal/ubiquitous or subclonal/heterogenous events in each gene across all samples. Metastatic hormone naive prostate cancer (mHNPC); high-risk localised prostate cancer (hrlPC).

**Figure 3.**
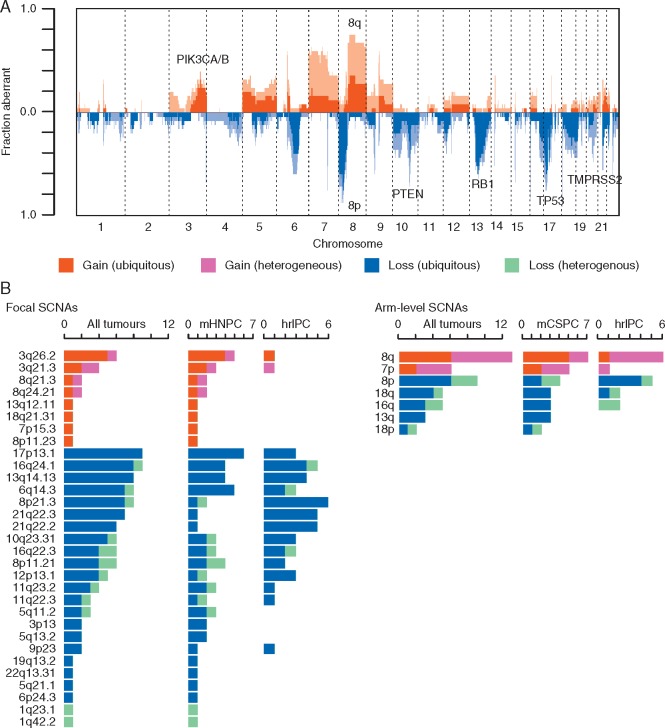
Recurrent somatic copy number alterations (SCNAs) in prostate cancer. (A) An overview of the SCNA landscape across all 25 tumours: fraction of cohort (*y*-axis) with ubiquitous gains (red), heterogeneous gains (pink), ubiquitous loss (dark blue) and heterogeneous loss (light blue) are shaded across the genome (*x*-axis). (B) Frequencies of occurrence of previously identified GISTIC focal and arm-level SCNAs across all tumours, metastatic on presentation (mHNPC) and non-metastatic on presentation (hrlPC) tumours. Shades of colours as in A.

To explore the relative frequency of SNVs and SCNAs in mHNPC and hrlPC, we focused on driver genes identified in previous PC series (Figure 2) [16, 17]. We found no significant differences between mHNPC and hrlPC tumours. However, there was a significant enrichment of 3q26.2 and 3q21.3 gains in mHNPC compared to hrlPC tumours (5/12 versus 1/13 and 3/12 versus 1/13, respectively) (Figure 3), which remained significantly enriched after controlling for the differing levels of SCNAs.

### Parallel evolution of wnt/β-catenin pathway

**Figure 4.**
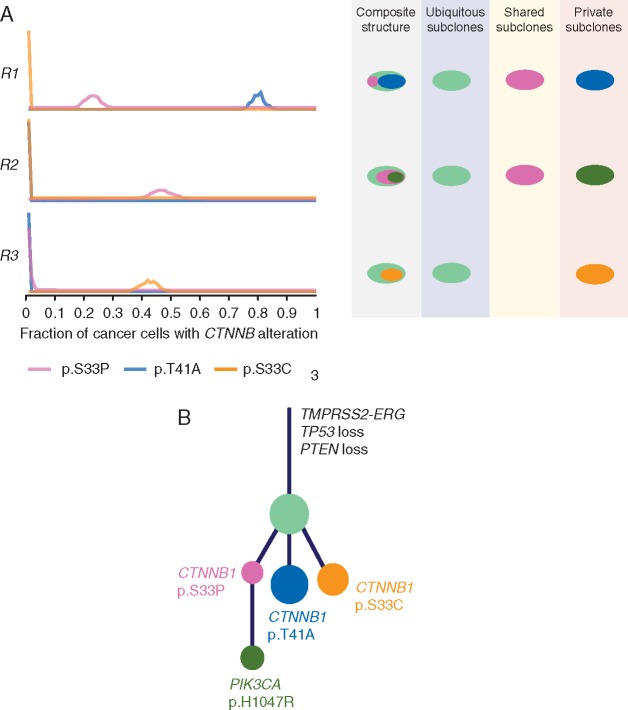
Parallel evolution in Wnt pathway in PR0139 and association with CD8^+^/FOXP3^+^ ratio across 15 tumours. (A) Left, fraction of cancer cells in sequenced tumour regions R1, R2 and R3 harbouring different *CTNNB1* mutations, p.S33P (pink), p.T41A (blue) and p.S33C (orange). Right, schematic showing different compositions of subclones in each sequenced tumour region, colours correspond to left panel. (B) Phylogenetic tree showing evolutionary history of PR0139 and acquisition of various driver mutations. Relative sizes of circles correspond to number of SNV mutations in that mutational cluster.

We observed one tumour (PR0139) with three distinct *CTNNB1* mutations, all previously described gain-of-function mutations in exon 3 of *CTNNB1* leading to stabilisation of β-catenin and activation of Wnt/β-catenin signalling (Figure 4A). Phylogenetic analysis of the clonal structure in this tumour revealed that all 3 *CTNNB1* mutations were in three separate subclones (Figure 4B), providing strong evidence for parallel evolution leading to activation of the Wnt/β-catenin pathway in this tumour.

### Temporal order of driver events in clinically high-risk PC

To explore the relative timing of driver events in PC, we utilised a modified version of Pyclone to cluster the mutations (supplementary Methods, available at *Annals of Oncology* online ). Consistent with previous reports about PC tumourigenesis [18, 19], we observed ETS fusions and mutations or loss of *TP53* to be early (clonal) events (Figure 2 and supplementary Table S3, available at *Annals of Oncology* online), *PTEN* a later event (60% clonal), and mutations or deletions of chromatin modifiers (*KMT2C, KMT2D and CHD1*) as a later (subclonal) event (Figure 2).

The landscape of SCNAs was also highly consistent with previous studies [15, 17], (Figure 3). In general, we observed that the majority of recurrent SCNA peaks were early events across most tumours in the cohort, aside from 8q and 7p gains, which occurred heterogeneously in 7/13 and 4/6 tumours.

Next, we investigated the mutational processes in the two patients with defective MMR (supplementary Figure S7, available at *Annals of Oncology* online). BP0001, who had germline *MSH2* and *MSH6* aberrations, had a high proportion of ubiquitous mutations associated with Signature 6 (DNA repair) compared with PR0103 (41.3% versus 8.6%) in keeping with loss of MMR as an early tumourigenic process. Conversely, ubiquitous mutations in PR0103 were mainly associated with Signature 1 (age), suggesting that MMRD was not an initial driver of this tumour, but rather the acquired biallelic loss of *MSH2* was a later event that provided a selective advantage, possibly through an accelerated mutation rate.

### T-cell infiltrate heterogeneity and neoantigen burden

**Figure 5.**
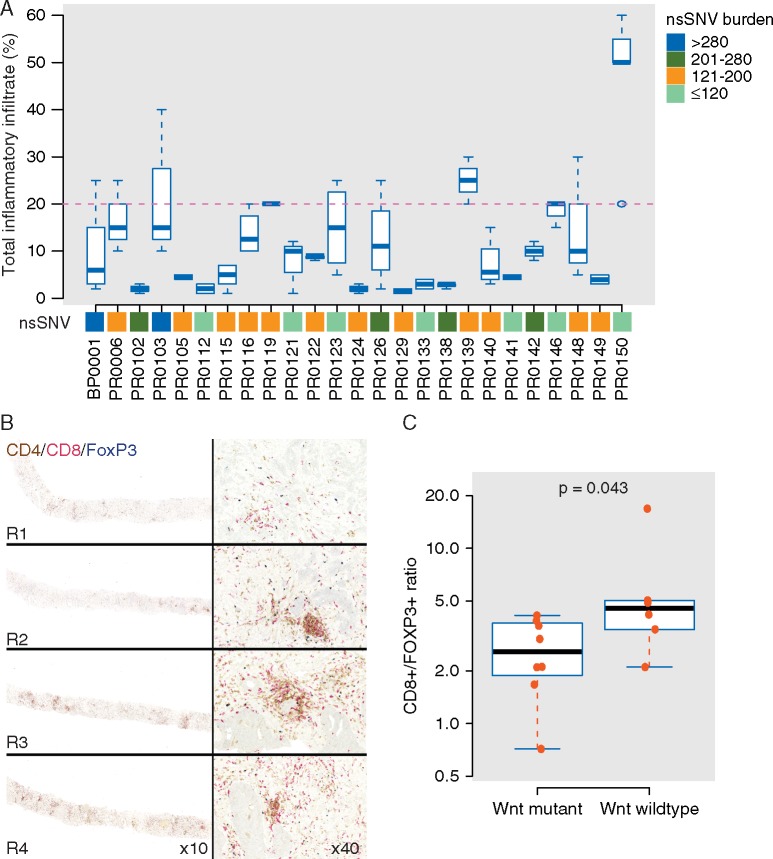
T-cell heterogeneity in prostate tumours. (A) Manual quantification of inflammatory infiltrate. Mean is represented by horizontal lines, box and whiskers show the 95% confidence interval and range, respectively. The dotted line marks the threshold for high inflammatory infiltrate. (B) Multiplex immunohistochemistry (IHC) analysis of four different prostate core biopsies (R1–4) from a patient, PR0123, showing heterogeneity in T-cell infiltration. CD8 staining in red, CD4 in brown and FoxP3 in blue. (C) Boxplot comparing CD8^+^/FOXP3^+^ ratios between tumours with and without somatic activation of Wnt pathway (gain-of-function mutation in *CTNNB1*, amplification in *RSPO2*, loss in *APC, RNF43* and *ZNRF3*) across 15 tumours with digital image analysis.

There was considerable variation in the total inflammatory infiltrate (INIF) (CD8^+^ or CD4^+^ and/or FoxP3^+^ cells in tumour region) between patients (Figure 5A), as well as between different regions within each patient. This intratumoural heterogeneity of INIF is well illustrated by PR0123, where 4 separate core biopsies have different levels of INIF (mean 15%, range 5%–25%) (Figure 5B).

We noted that both PR0103 and BP0001 had extensive INIF (maximal infiltrate >20% of all nucleated cells per biopsy) (2/2) compared with patients without MMR deficiency, where only 6/23 had extensive INIF. Patients PR0112 and PR0129 had ubiquitous and heterozygous loss of *MLH1* and *MSH2* respectively, but this was not associated with high mutational burden or high INIF. As mutational load has been reported to correlate with neoantigen load and neoantigens can elicit a clonal expansion of neoantigen reactive T- (NART) cells [20–22], we hypothesised that the abundant INIF in these MMRD deficient tumours might be related to a high neoantigenic burden. Consistent with this, PR0103 and BP0001 displayed a high neoantigen burden. However, extending this analysis to all 25 patients in this cohort, there was no association between neoantigen burden nor clonal neoantigen burden and INIF (supplementary Figure S8, available at *Annals of Oncology* online).

### Wnt signalling and modulation of immune response

Activation of tumour intrinsic Wnt/β-catenin signalling in melanoma has recently been reported to lead to T-cell exclusion from the tumour preventing anti-tumour immunity [23]. However, PR0139 who had parallel evolution of activated β-catenin, had high levels of CD8^+^ infiltrate (Figure 5A), but was noted to also have high FOXP3^+^ levels giving a low CD8^+^/FOXP3^+^ ratio. A low ratio of tumour-infiltrating CD8^+^ and FOXP3^+^ lymphocytes is increasingly being recognised as a measure of immune suppression and as a potential prognostic indicator [24–26].

In our cohort, the 15 patients with levels of INIF at or above the median underwent digital pathology analysis. Of these patients, 7/15 had activating mutations in the Wnt pathway (gain-of-function *CTNNB1* mutations, *RSPO2* amplification, and deletion of *APC, RNF43* and *ZNRF3* [17, 27]). We observed a significantly lower CD8^+^/FOXP3^+^ ratio in patients with tumours containing activating mutations of the Wnt pathway compared with wild-type tumours (2.65 ± 1.2 versus 6.08 ± 5.0, *P *= 0.043, Mann–Whitney *U* test) (Figure 5C).

## Discussion

We have conducted the largest prospective clinical cohort study of M-Seq in high-risk PC patients and carried out an integrated genomic and tumour immune infiltrate analysis. Uniquely, we have compared M-Seq of diagnostic prostate biopsies from mHNPC and hrlPC and demonstrated increased SCNA in mHNPC patients, consistent with previous reports correlating biochemical recurrence following prostatectomy with high SCNA in localised disease [28]. We observed no differences in SNV frequency between mHNPC and hrlPC patients, which is surprising given the large differences seen in other studies between localised PC and pre-treated metastatic castrate-resistant prostate cancer [17, 29]. This may be a consequence of the small sample size or may suggest that unlike SCNA changes, SNV accumulation is a later evolutionary event, possibly as a result of the selective pressure of treatment. In this study, there was enrichment for gains of 3q26.2 and 3q21.3 in mHNPC patients. Both amplicons contain genes previously implicated in PC, e.g. 3q26.2 contains *PRKCI*, expression of which is associated with biochemical relapse following prostatectomy [30]. Interestingly, these gains in copy number are early evolutionary events, and the fact that these focal gains are enriched in patients presenting with metastatic disease suggests that some PCs are hard-wired to be aggressive.

We describe the first report of parallel evolution of Wnt signalling in PC, where 3 separate gain-of-function mutations of β-catenin (*CTNNB1*) were identified in a single tumour. This is similar to the distinct *TMPRSS-ERG* fusions identified in several regions of the primary prostate tumour [5] and alterations of *SETD2*, *PTEN* and *KDM5C* in renal cancer [31]. Parallel evolution of the Wnt pathway, a pathway already implicated in PC cell growth, proliferation and epidermal to mesenchymal transition [32], points to its biological importance in PC. Unlike mouse melanoma models, where tumour intrinsic Wnt/β-catenin signalling led to T-cell exclusion from the tumour [23], we observed that patients with activated Wnt/β-catenin signalling can have normal or high levels of INIF, but that this is predominantly CD8^+^/FOXP3^+^ low, consistent with a dysfunctional T-cell response. Future studies will be needed to further elucidate the role and mechanism of Wnt/β-catenin signalling in immune modulation in human PC, which is of particular interest given the number of potential novel drugs targeting this pathway.

We identified two patients with hypermutation associated with MMR deficiency and high INIF, the latter being similar to a report of 12/16 (75%) men at risk of Lynch syndrome and diagnosed with PC having significant INIF [33]. Similar to reports in advanced PC [34], our hrlPC patients with MMRD had complex structural rearrangements of DNA repair genes *MSH2* and *MSH6* leading to inactivation. Overall however, we did not demonstrate an association with INIF and neoepitope burden, but given the small number of patients with DNA repair aberrations in this series, this analysis is underpowered. MMRD deficiency has been associated with response to immune checkpoint inhibition in a number of tumour types including PC [35, 36]. The finding of high INIF and neoepitope burden in some PC patients in this study supports current attempts to evaluate the role of mutational burden and neoepitopes in prospective therapeutic clinical trials (NCT02113657 and NCT03061539).

We have demonstrated extensive intratumoural heterogeneity of INIF in primary PC. The impact of this on prognosis and predicting treatment response is unknown, but future studies testing INIF as a potential biomarker will need to consider testing multiple tumour regions or developing a liquid biopsy strategy.

In conclusion, our findings reveal how mutational and SCNA changes may drive aggressive metastatic PC. We show that activated Wnt signalling is correlated with immune suppression in primary PC, and suggest that activated Wnt/β-catenin, MMR, high INIF and the CD8^+^/FOXP3^+^ ratio should be explored as predictive biomarkers for immunotherapeutics in prostate cancer.

## Funding

This work was supported by Prostate Cancer Foundation. CS, ML, ME, SAQ and TS are supported by the National Institute for Health Research, the University College London Hospitals Biomedical Research Centre (no grant numbers apply). ML has received support from a BMS II-ON grant (no grant numbers apply). ME is a UK National Institute of Health Research (NIHR) Senior Investigator. CS, HUA and ML are supported by the Cancer Research UK University College London Experimental Cancer Medicine Centre (no grant numbers apply). CS is Royal Society Napier Research Professor. This work was supported by the Francis Crick Institute (no grant numbers apply) which receives its core funding from Cancer Research UK (FC001169), the UK Medical Research Council (FC001169), and the Wellcome Trust (FC001169); by the UK Medical Research Council (grant reference MR/FC001169/1); CS is funded by Cancer Research UK (TRACERx), the CRUK Lung Cancer Centre of Excellence, Stand Up 2 Cancer (SU2C), the Rosetrees Trust, NovoNordisk Foundation (ID 16584), the Prostate Cancer Foundation, the Breast Cancer Research Foundation (BCRF), the European Research Council (THESEUS) (no grant numbers apply). HUA acknowledges funding from the Medical Research Council (UK), the Pelican Cancer Foundation Charity, Prostate Cancer UK, St Peters Trust Charity, Prostate Cancer Research Centre the Wellcome Trust, National Institute of Health Research-Health Technology Assessment Programme and the US National Institute of Health-National Cancer Institute (no grant numbers apply). SAQ is funded by a CRUK Career Development Fellowship, CRUK Biotherapeutic Programme Grant, World Wide Cancer Research, and a Cancer Research Institute Investigator Award (no grant numbers apply). ZS is supported by the Breast Cancer Research Foundation, Basser Foundation, Mazzone Foundation, EU FP7 project PREDICT, the Széchenyi Progam, Hungary (KTIA_NAP_13-2014-0021) and the NovoNordisk Foundation (ID 16854). The funders had no role in study design, data collection and analysis, decision to publish, or preparation of the article. 

## Disclosure

The authors have declared no conflicts of interest.

Key Message

Multiregion analysis of high-risk prostate cancer showed intratumoural heterogeneity of INIF, mutations and copy number. INIF was associated with hypermutation due to mismatch repair (MMR) deficiency and low CD8^+^/FoxP3^+^ correlated with activating mutations in the Wnt pathway. INIF, Wnt/βcatenin and MMR should be explored as immunotherapy predictive biomarkers.

## Supplementary Material

Supplementary Figure S1

Supplementary Figure S2

Supplementary Figure S3

Supplementary Figure S4

Supplementary Figure S5

Supplementary Figure S6

Supplementary Figure S7

Supplementary Figure S8

Supplementary Table S1

Supplementary Table S2

Supplementary Table S3

Supplementary Methods
