# Supplementary figures and images for: Intratumoural evolutionary landscape of high-risk prostate cancer: the PROGENY study of genomic and immune parameters
Source: Ann Oncol. 2017 Jul 19;28(10):2472–80. doi: 10.1093/annonc/mdx355 (PMC5815564; doi:10.1093/annonc/mdx355)

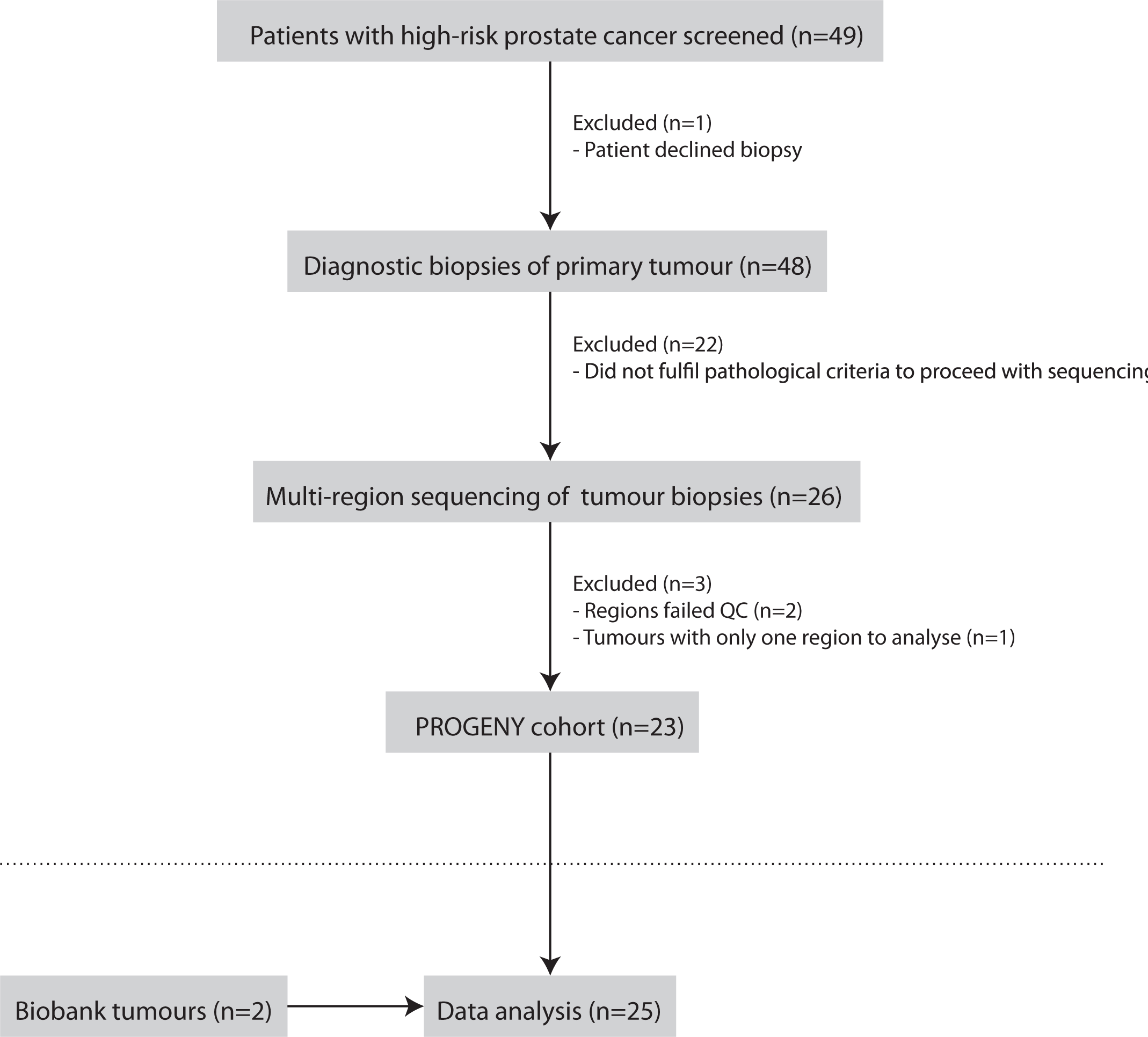

Supplement: Supplementary Figure S1 [file fig_s1_consort2_mdx355.png]

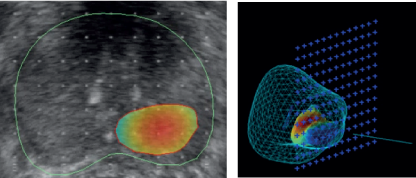

Supplement: Supplementary Figure S2 [file fig_s2_mri_image2_mdx355.png]

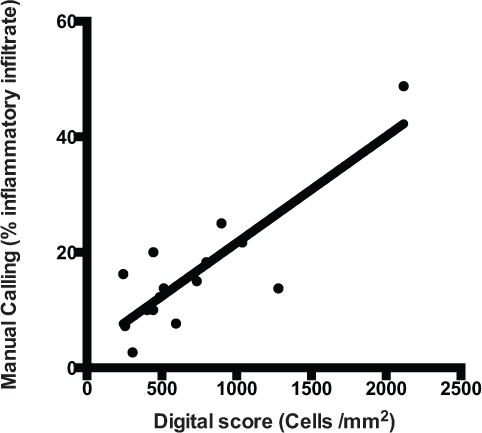

Supplement: Supplementary Figure S3 [file fig_s3_manual_digital2_mdx355.png]

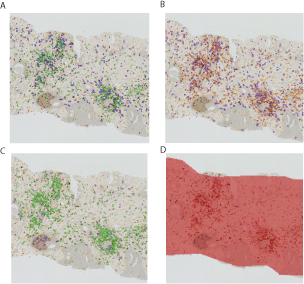

Supplement: Supplementary Figure S4 [file fig_s4_digital_image_analysis2_mdx355.png]

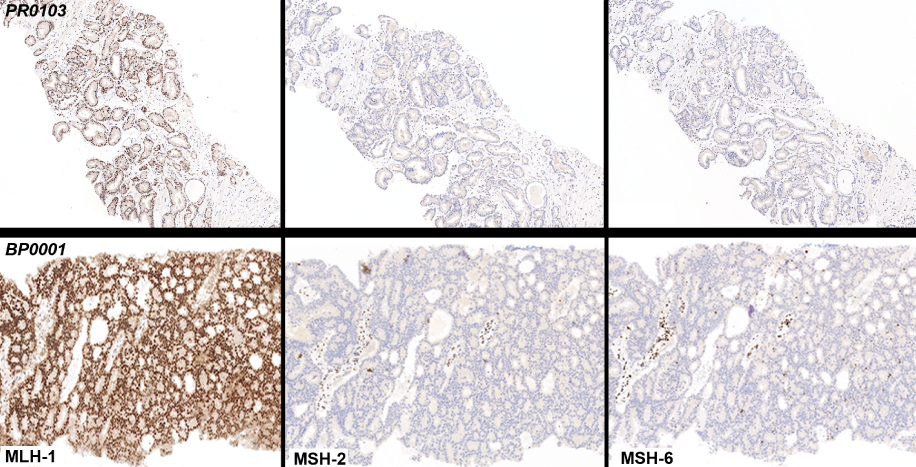

Supplement: Supplementary Figure S5 [file fig_s5_mmr_ihc2_mdx355.png]

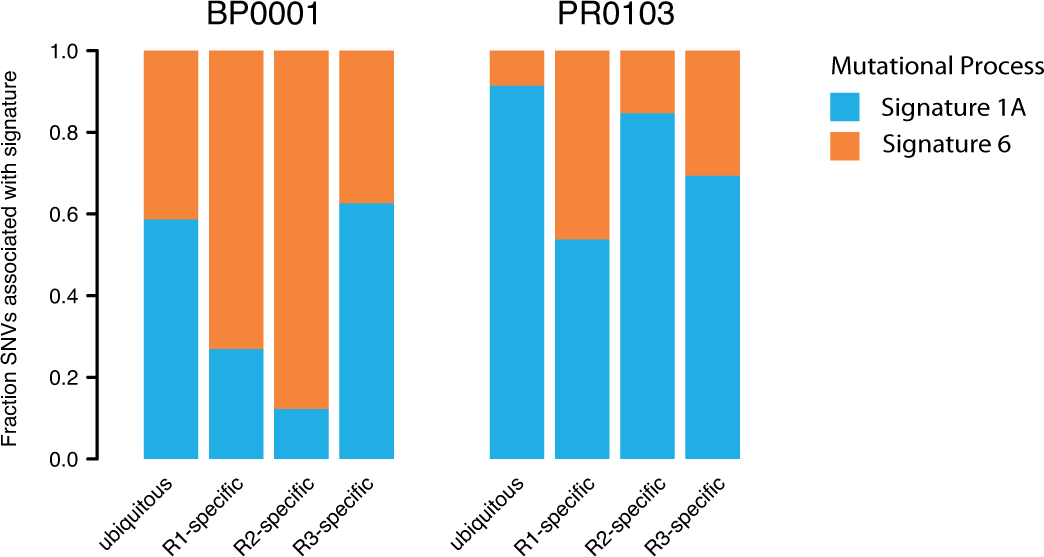

Supplement: Supplementary Figure S6 [file fig_s6_hyper_signatures2_mdx355.png]

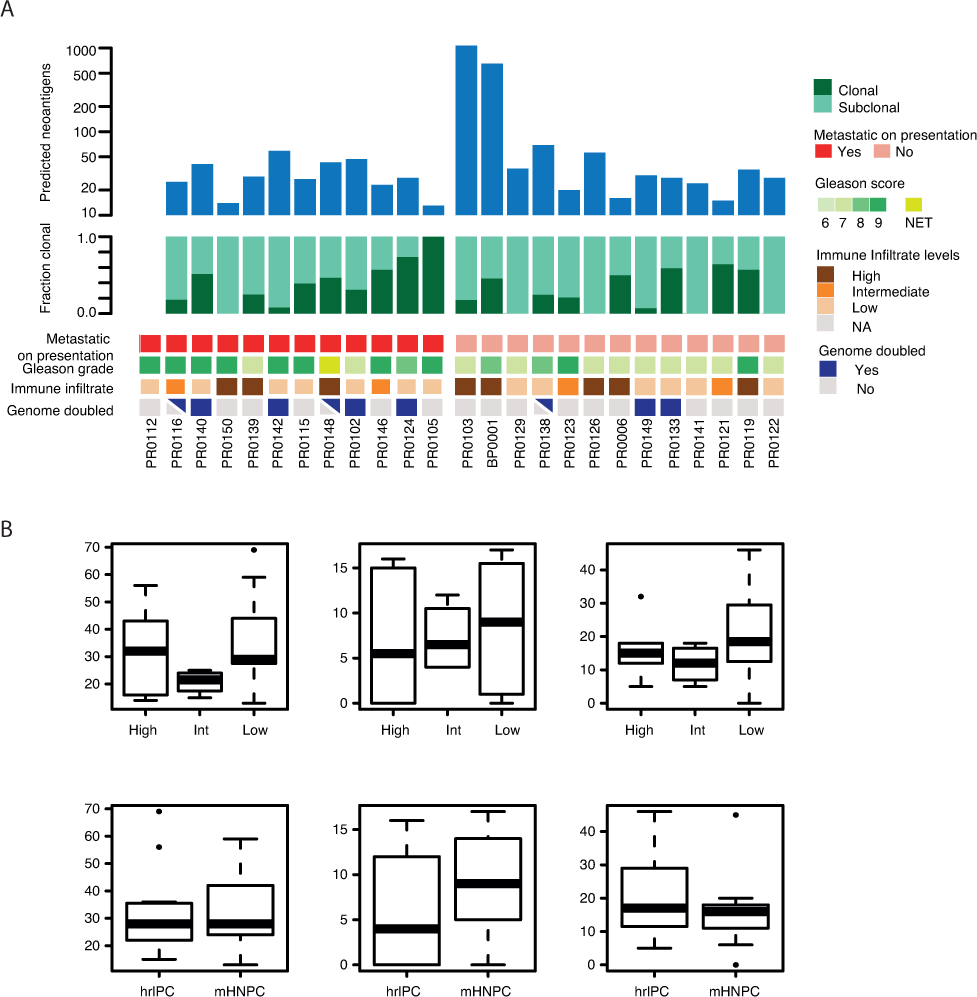

Supplement: Supplementary Figure S7 [file fig_s7_neoantigens2_mdx355.png]

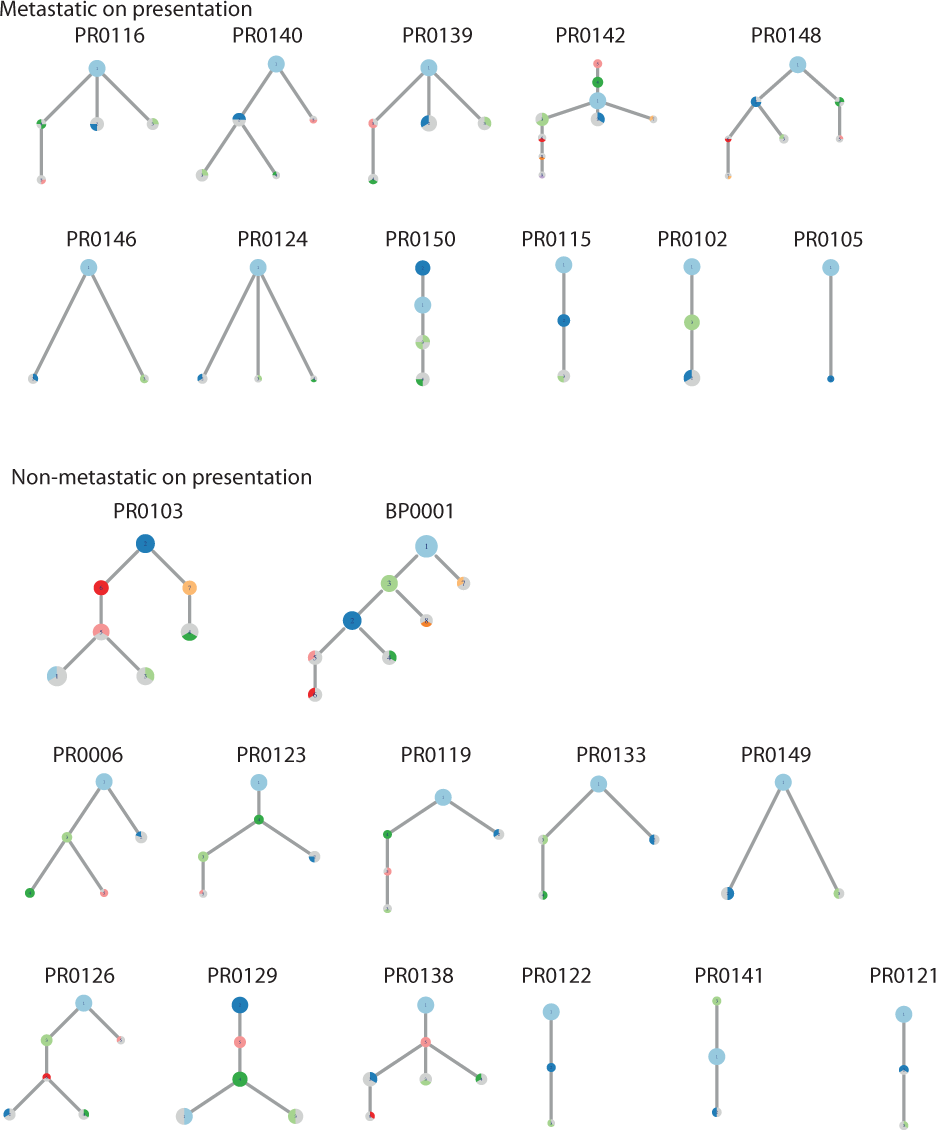

Supplement: Supplementary Figure S8 [file fig_s8_trees2_mdx355.png]
