# Supplementary material for: Intratumoural evolutionary landscape of high-risk prostate cancer: the PROGENY study of genomic and immune parameters
Source: Ann Oncol. 2017 Jul 19;28(10):2472–80. doi: 10.1093/annonc/mdx355 (PMC5815564; doi:10.1093/annonc/mdx355)
Supplement: Supplementary Table S1 [file progeny_table_s1_mdx355.docx]

**Table S1. Prostate cancer characteristics of Progeny patients**

| Patient ID | Age | PSA | MRI stage | M-stage | Gleason score | Grade Grouping | Multifocal | No. cores |
| --- | --- | --- | --- | --- | --- | --- | --- | --- |
| PR0006 | 54 | 11 | T3bN0 | M0 | 4+3 | 3 | No | 3 |
| PR0102 | 78 | 650 | T3bN0 | M0 | 4+3 | 3 | No | 3 |
| PR0103 | 76 | 73 | T3aN0 | M0 | 4+3 | 3 | No | 3 |
| PR0105 | 63 | 48 | T3bN1 | M1 | 4+5 | 5 | No | 2 |
| PR0112 | 71 | 20 | T3bN1 | M1 | 4+5 | 5 | No | 3 |
| PR0115 | 73 | 120 | T3bN1 | M1 | 4+5 | 5 | No | ­­­­­5 |
| PR0116 | 72 | 29 | T4N1 | M1 | 5+4 | 5 | Yes | 4 |
| PR0119 | 73 | 32 | T3bN0 | M0 | 4+5 | 5 | No | 3 |
| ­­­­PR0121 | 78 | 17 | T3aN0 | M0 | 4+3 | 3 | No | 3 |
| PR0122 | 76 | 60 | T3aN0 | M0 | 3+4 | 2 | No | 3 |
| PR0123 | 77 | 26 | T3bN0 | M0 | 4+5 | 5 | No | 4 |
| PR0124 | 77 | 47 | T3bN1 | M1 | 4+4 | 4 | No | 3 |
| PR0126 | 80 | 9 | T3aN0 | M0 | 3+4 | 2 | Yes | 3 |
| PR0129 | 69 | 12 | T3aN0 | M0 | 3+4 | 2 | No | 2 |
| PR0133 | 86 | 32 | T3bN0 | M0 | 4+3 | 3 | No | 2 |
| PR0138 | 82 | 35 | T2bN0 | M0 | 4+3 | 3 | No | 3 |
| PR0139 | 73 | 65 | T3bN1 | M1 | 3+4 | 2 | No | 3 |
| PR0140 | 82 | 112 | T3bN0 | M1 | 4+5 | 5 | No | 4 |
| PR0141 | 70 | 32 | T2cN0 | M0 | 3+4 | 2 | No | 3 |
| PR0142 | 77 | 71 | T4N1 | M1 | 4+5 | 5 | No | 3 |
| PR0146 | 58 | 65 | T2bN1 | M1 | 4+5 | 5 | No | 3 |
| PR0148 | 78 | 19 | T4N1 | M1 | 5+5* | 5 | No | 4 |
| PR0149 | 64 | 15 | T2bN0 | M0 | 4+3 | 3 | No | 3 |
| PR0150 | 68 | 59 | T3bN1 | M1 | 4+5 | 5 | No | 4 |
| BP0001 | 52 | 171 | T3aN0 | M0 | 4+5 | 5 | No | 3 |

PSA, prostate specific antigen; MRI, magnetic resonance imaging; M-stage, metastasis-stage; Multifocal, fulfills criteria for multifocality on MRI criteria; * Neuroendocrine tumour.
