# Supplementary material for: Intratumoural evolutionary landscape of high-risk prostate cancer: the PROGENY study of genomic and immune parameters
Source: Ann Oncol. 2017 Jul 19;28(10):2472–80. doi: 10.1093/annonc/mdx355 (PMC5815564; doi:10.1093/annonc/mdx355)
Supplement: Supplementary Table S2 [file progeny_table_s2_mdx355.docx]

**Table S2. Primary antibodies**

| **Molecule** | **Antibody type** | **Clone name** | **Dilution** | **Source** |
| --- | --- | --- | --- | --- |
| Anti-Human  B-Catenin | Mouse Monoclonal | 17C2 | 1:100 | Leica Biosystems Newcastle Ltd, UK |
| Anti-human CD4 | Rabbit Monoclonal | SP35 | 1:25 | Spring Biosciences Inc., Pleasanton, CA, US |
| Anti-human CD8 | Rabbit Monoclonal | SP239 | 1:100 | Spring Biosciences Inc., Pleasanton, CA, US |
| Anti-human FOXP3 | Mouse Monoclonal | 236A/E7 | 1:100 | Kind gift from  Dr G Roncador, CNIO, Madrid (Spain) |
| Anti-Human  MLH1 | Mouse Monoclonal | ES05 | 1:200 | Leica Biosystems Newcastle Ltd, UK |
| Anti-Human  MSH2 | Mouse Monoclonal | FE11 | 1:50 | Dako UK Ltd, Ely Cambridgeshire, UK |
| Anti-Human  MSH6 | Rabbit Monoclonal | EP49 | 1:50 | Dako UK Ltd, Ely Cambridgeshire, UK |
