# Supplementary material for: Intratumoural evolutionary landscape of high-risk prostate cancer: the PROGENY study of genomic and immune parameters
Source: Ann Oncol. 2017 Jul 19;28(10):2472–80. doi: 10.1093/annonc/mdx355 (PMC5815564; doi:10.1093/annonc/mdx355)
Supplement: Supplementary Table S3 [file progeny_table_s3_mdx355.docx]

**Table S3. Breakpoints of ETS fusions detected in 9 prostate cancers.**

| **Patient** | **Region** | **Chr** | **Pos** | **Chr** | **Pos** | **SV type** | **Genes** |
| --- | --- | --- | --- | --- | --- | --- | --- |
| PR0116 | R1,R2,R3,R4 | chr21 | 39862204 | chr21 | 42872985 | DEL | ERG-TMPRSS2 |
| PR0119 | R1,R2,R3 | chr21 | 39873721 | chr21 | 42873813 | DEL | ERG-TMPRSS2 |
| PR0121 | R1,R2,R3 | chr21 | 39860994 | chr21 | 42868005 | DEL | ERG-TMPRSS2 |
| PR0122 | R1,R2,R3 | chr21 | 39868743 | chr21 | 42867540 | DEL | ERG-TMPRSS2 |
| PR0139 | R1,R2,R3 | chr21 | 39872508 | chr21 | 42840167 | INV | ERG-TMPRSS2 |
| PR0140 | R1,R2,R3,R4 | chr21 | 39934287 | chr21 | 42876327 | INV | ERG-TMPRSS2 |
| PR0123 | R1,R2, R3,R4 | chr21 | 39830087 | chr21 | 42868498 | DEL | ERG-TMPRSS2 |
| PR0133 | R1, R2 | chr21 | 39836998 | chr21 | 42862587 | DEL | ERG-TMPRSS2 |
| PR0006 | R1 | chr21 | 42874875 | chr7 | 14020612 | TRA | TMPRSS2-ETV1 |
|  | R2 | chr21 | 42874909 | chr7 | 14020618 | TRA | TMPRSS2-ETV1 |
|  | R3,R5 | chr21 | 42874207 | chr7 | 14020423 | TRA | TMPRSS2-ETV1 |
|  | R4 | chr21 | 42874910 | chr7 | 14020617 | TRA | TMPRSS2-ETV1 |
