# Supplementary material for: Intratumoural evolutionary landscape of high-risk prostate cancer: the PROGENY study of genomic and immune parameters
Source: Ann Oncol. 2017 Jul 19;28(10):2472–80. doi: 10.1093/annonc/mdx355 (PMC5815564; doi:10.1093/annonc/mdx355)
Supplement: Supplementary Methods [file progeny_supp_revisions_clear_mdx355.docx]

**Supporting Information**

**Supplementary Methods**

**Patient information**

Men with an elevated prostate specific antigen (PSA), with no prior biopsy, no prior systemic or local treatment for prostate cancer and no prior pelvic radiotherapy for other cancers were eligible provided they had undergone a multi-parametric MRI according to international guidelines showing at least one lesion scoring Prostate Imaging Reporting and Data System (PIRADS) 4 or 5 with a largest diameter of more than 1cm in the axial. The 1cm threshold in the axial plane was set as the transperineal template image-fusion sampling was carried out every 5mm through a standardized brachytherapy grid placed against the perineum and we required at least two regions to be sampled from the largest lesion in order to investigate ITH. No upper limits were placed on age, TNM stage or PSA. Classification of patients as metastatic hormone naive prostate cancer (mHNPC) or high risk localized prostate cancer (hrlPC) was radiologically determined using bone-scan and/or Choline PET-CT and was independent of the PSA.

For the analysis we included a further 2 patients where the patient samples were obtained as part of our institutional biobank consent (REC 15/YH/0311). PR0006 was a pilot patient prior to PROGENY study initiation to assess our processes and operating procedures. The multiregion fresh samples were obtained from core biopsy of the prostate specimen following radical prostatectomy and prior to formalin fixation, using established methods [1]. BP0001 was a patient consecutive to the PROGENY cohort with treatment naïve prostate cancer undergoing a routine Multiparametric (MP)-MRI directed multi-region template biopsy. A previous prostate biopsy made him ineligible for PROGENY, therefore fresh frozen samples at the time of repeat biopsies were taken within the context of our institutional biobank consent and processed using the study protocols in parallel.

For this genetic and T-cell analysis, we focused predominantly on the patients with: D’Amico defined clinically high risk prostate cancer (PSA ≥20ng/ml, stage≥T2c and Gleason 8-10), MP-MRI of sufficient quality to perform textural analysis (reported elsewhere); ≥30% tumour content within ≥2 biopsies; high quality sequencing data.

**Tissue procurement and processing**

Two biopsy cores were taken from each assigned transperineal biopsy grid reference (referred to as a region) using a Bard Max Core® 18Gauge 20mm core length needle; one for genomic analysis and the other for morphology and immunohistochemistry. The first sample was fixed in 10% formaldehyde and transported to the routine diagnostic laboratories for morphology and immunohistochemistry. The second spatially defined biopsy was wrapped in aluminium foil (VWR International #293-4113), snap frozen in liquid nitrogen and then placed in 2ml cryogenic vial (Corning #430659) and stored at -80^o^C. The percentage biopsy tumor content of the snap frozen genomic sample was estimated from the corresponding FFPE sample. A cut off of ≥30% biopsy tumor content, as assessed by a uro-pathologist, in at least 2 regions was selected to attain good DNA yield. Extracted DNA quantity was checked by spectrophotometry (NanoDrop 1000, Thermoscientific) and the quality was assessed by gel electrophoresis.

**Multiparametric Prostate Magnetic Resonance Imaging**

MP-MRI was performed prior to study consent on a range of 5 machines at 1.5 and 3 Tesla (T) with 2 manufacturers (Philips, Best, the Netherlands and Siemens, Erlangen, Germany), using pelvic phased array coils. 0.2mg/kg (up to 20mg) of a spasmolytic agent (Buscopan; Boehringer Ingelheim, Germany) was administered intravenously prior to imaging to reduce bowel peristalsis. MP-MRI comprised axial and coronal T2 turbo spin echo (TSE) imaging, supplemented with diffusion-weighted imaging at 4 b-values (0,150, 500 and 1000 s/mm2) from which an apparent diffusion coefficient (ADC) map was calculated. A separate high b-value acquisition was also carried out for improved signal-to-noise ratio for lesion detection purposes (b1400 at 1.5T and b2000 at 3T). A dynamic contrast enhanced (DCE) acquisition was subsequently performed using spoiled gradient echo with fat saturation and a 12 second time resolution. 0.2ml/kg intravenous macrocyclic gadolinium-based contrast agent (Prohance, Bracco, Milan, IT) was injected at the beginning of the 6^th^ acquisition at 3ml/s followed by a 20ml saline. All acquisitions were compliant with UK (17) and European (18) guidelines.

**Immunohistochemistry**

Tumour samples and reactive human tonsils were fixed in 10% buffered formalin and embedded in paraffin according to conventional histological protocols. 2-5μm tissue sections were cut and transferred onto poly-l-lysine–coated slides, dewaxed in two changes of xylene and rehydrated in a series of graded alcohols. Single immunohistochemistry was carried out using the automated platforms BenchMark Ultra (Ventana/Roche) and the Bond-III Autostainer (Leica Microsystems) according to a protocol described elsewhere [2, 3]. To establish optimal staining conditions (i.e. antibody dilution and incubation time, antigen retrieval protocols, suitable chromogen) each antibody was tested and optimized on sections of human reactive tonsil, used as positive control.

Multiplex immunohistochemistry was carried out using a protocol described previously [4]. For evaluation of protein co-expression in the cytoplasm and cell membrane, change of the single colour of the chromogen was noted, i.e. blue and red gave rise to a purple and brown and blue to an almost black labelling. Co-expression of nuclear and cytoplasmic or membranous proteins was easy to detect, as the colour of the chromogens remained distinct. Specificity of the staining was assessed by a haematopathologist (TM) with expertise in multiplex-immunostaining. Slides were scanned using the Hamamatsu Nanozoomer Digital scanner.

**Digital Image analysis**

For the automatic image analysis, segmentation of CD4+ and FOXP3+ cells was performed using the Definiens Tissue Phenomics software platform utilizing a previously described machine learning-based parameter-free cell segmentation [5]. Next, CD8+ cells were segmented and both results combined. Finally, the results were post-processed for accurate splitting of cells using a watershed transform based on the distance map of the segmentation result weighted with the original gray values of the source image (supplementary Figure S4). Following this, the cell segmentation results were combined with a binary mask of the tumor region obtained by manual annotation of a human expert to discard regions outside of the tumor. For quantitative evaluation, the number of cells per area was determined.

**Multi-region whole-genome and whole-exome sequencing**

Whole-genome sequencing was performed using 1-2 μg DNA isolated from regions from four tumours (PR0006, PR0102, PR0103, PR0105) and matched germline genomic DNA by Illumina Cambridge Ltd. to a median coverage depth of 106x and 39x, respectively. Whole-exome sequencing was carried out on the remaining 21 tumours and matched germline genomic DNA using a customised version of the Agilent SureSelect V6 exome according to the manufacturer’s protocol (Agilent). The capture kit was extended to cover intronic regions in genomic areas linked with fusion events in prostate cancer (TMPRSS2, ERG, SLC45A3, ETV1, ETV4, ELK4 HNRNPA2B1). Sequencing was performed by AROS Applied Biotechnology to a median coverage depth of 199x and 115x in tumour regions and germline, respectively.

**Genomic analysis**

*Variant calling*

The generated raw sequencing data was aligned to the human reference genome GRCh37/hg19 with bwa (v0.5.9) [6], duplicates marked using Picard (v1.54), and indel realignment with GATK IndelRealigner (v1.0.6076) [7]. Exonic regions from the whole-genome genome data were extracted using the exome capture design bedfile to ensure uniform downstream analysis all samples.

Somatic single nucleotide variant (SNV) calling and insertions and deletion (indel) calling were performed using VarScan2 (v2.3.7) [8], and a minimum variant allele frequency (VAF) of 5% was used as a threshold. In cases where a somatic variant was not ubiquitously called across all tumour regions, the coverage depth across all regions was extracted using bam-readcount (v0.5.1) (https://github.com/genome/bam-readcount) and SAMtools mpileup (v0.1.19) [9], thereby enabling assessment of the variant in all regions, and the VAF threshold was lowered to 1%. Variants were annotated using Annovar (version 2014Jul14) [10].

Somatic copy number aberrations (SCNAs), ploidy and cellularity were determined from processed exome copy number data generated with VarScan2 (v2.3.6) using ASCAT2 [11](available at <https://github.com/Crick-CancerGenomics/ascat>). Manual verification was performed of the automatically selected models for ploidy and cellularity. Copy number segments were defined relative to ploidy as previously [12]: amplification, gain and loss were defined as log2(4/2), log2(2.5/2) and log2(1.5/2), respectively.

Genome-doubling status was determined as previously described [13]. Briefly, each sample, s, was represented as an aberration profile of major and minor allele copy numbers at chromosome arm resolution. The total number of aberrations (relative to diploid), Ns, and the probabilities of loss/gain for each allele at each chromosome arm, Ps, was calculated. Ten thousand simulations were run for each sample s, where Ns sequential aberrations, based on Ps, were applied to a diploid profile. A *p*-value for genome doubling was obtained by counting the percentage of simulations in which the proportion of chromosome arms with a major allele copy number ≥2 was higher than that observed in the sample.

Structural variant (SV) calling was performed with DELLY2 (v0.7.2) [14]. Somatic calls were made using the somatic filter provided. ETS fusions were manually reviewed using Integrative Genomics Viewer [15].

*Neoantigen calling*

In order to identify neoantigens, we first performed HLA typing with OptiType [16]. Nonsynonymous mutations were extracted from each tumour sample and translated into a mutant peptide between 9-11mers long. Using the patient-specific HLA type, we utilized NetMHC (v2.8) [17] to predict the binding strength of each mutant and wildtype peptide to the respective MHC class I molecules. Somatic mutations that gave rise to peptides with a binding affinity of ≤500nM were considered to be putatively neoantigenic. Neoantigens were further subdivided into strong (<50nM) or weak binders (50nm-500nM), and clonal or subclonal depending on the status of the somatic mutation.

*Subclonal dissection*

In order to estimate whether somatic SNVs and indels were clonal (occurring earlier) or subclonal (occurring later), and to elucidate the clonal structure of each tumor, a modified version of PyClone [18] was used. Full details of this modified version can be found at <https://bitbucket.org/nmcgranahan/clonalneoantigenanalysispipeline> [19]. Briefly, the mutation copy number and cancer cell fraction of each mutation were calculated by integrating ASCAT-derived integer copy number and tumour purity estimates with the variant frequency. This was used as input for PyClone, which uses a hierarchical Bayesian Dirichlet process in order to infer clonal population structure.

To determine if SCNAs were ubiquitous or heterogeneous, we considered all parts of the genome independently and split into minimum consecutive segments of overlap within each tumour across all regions. Segments of gain or loss that overlapped across all regions was defined as ubiquitous and all other segments of SCNA as heterogeneous.

*Identification and timing of driver events*

A list of significantly mutated genes in prostate cancer was compiled from [20] and [21]. Clonal status of somatic mutations and copy number status of these genes were integrated for this analysis. GISTIC focal regions and arm-length events were considered using a threshold of <80% and ≥80% of the chromosomal arm, respectively.

*Phylogenetic tree construction*

To infer phylogenetic relationship between clusters of mutations, a one sided t-test was performed between the cancer cell fraction of each mutation cluster pair, across all tumour regions. Mutation clusters with at least 5 mutations assigned were included in the phylogenetic analysis. A p-value threshold of 0.025 was applied to determine if a given cluster of mutations was significantly smaller than another cluster. If cluster A was significantly smaller than B in at least one region, and cluster B is not significantly smaller than cluster A in any regions, then cluster A was determined to have descended from cluster B.

*Mutational signatures*

Mutational signatures were determined using the R package deconstructSigs (v1.4) [22]. Using this tool, the fraction of mutations in each of the 96 trinucleotide contexts was calculated, and the weighted combination of published signatures from [23] were identified to most closely reconstruct the mutational profile of the sample. Signatures 1A, 5, 6 and 8 were considered, based on previous reports [23, 24]. The two hypermutated samples (BP0001 and PR0103) were excluded from the aggregate mutational profile in the temporal analysis in order not to bias the profile towards Signature 6.

**Statistical analysis**

The Mann-Whitney U or Fisher’s exact tests were utilized when comparing between groups, as specified in the text. Multiple-testing corrections were performed using the Benjamini-Hochberg false discovery rate method. All values are represented as mean ± standard deviation, unless otherwise specified.

**Data availability**

All sequencing data is available through the European Genome-phenome Archive (EGA), accession number: EGAXXXX.

**Figure S1. CONSORT Diagram for patient recruitment into the PROGENY study.**

**Figure S2 Ultrasound image showing contoured prostate with prostatic lesion and overlaid template biopsy grid.** Locations for biopsy are selected on basis of mpMRI

**Figure S3. Comparison between manual and digital quantification of inflammatory infiltrate (R^2^=0.71)**

**Figure S4. Digital image analysis of CD4+ FOXP3+ and CD8+ cells.** A) CD4+ FOXP3+ cells. B) CD8+ cells. C) Combination and splitting of cells. D) Manual annotation by human expert.

**Figure S5. IHC staining of MMR proteins in hypermutant tumours.** Single MLH1/MSH2/MSH6 immunohistochemistry (IHC) analysis of a prostate core biopsy from PR0103 and BP0001 in 10X and 20X magnification respectively. Brown staining showing positivity in MLH1 and lack of brown staining showing loss of MSH2 and MSH6.

**Figure S6. Mutational processes in hypermutant tumours.** Mutational signatures 1A (blue, age associated) and 6 (orange, defective DNA mismatch repair) detected in BP0001 and PR0103 in ubiquitous mutations as well as region-specific mutations.

**Figure S7. Neoantigen heterogeneity in prostate cancer.** A) Barplot of total neoantigen burden across 25 tumours. No neoantigens were predicted in PR0112. Data tracks below indicate fraction of neoantigens that are clonal (dark green) and subclonal (light green), if patient was metastatic on presentation (red), Gleason grade (shades of green), level of tumoural inflammatory infiltrate (shades of brown), and if the tumour had undergone whole-genome doubling (purple, triangle indicating heterogeneous genome doubling). B) Boxplots comparing total (left), clonal (middle) and subclonal (right) neoantigen burden (y-axis) between tumours with varying immune infiltrate levels (top) and metastatic castrate sensitive prostate cancer (mHNPC) versus high risk localized prostate cancer (hrlPC) tumours. NMP, not metastatic at presentation (hrlPC); MP, Metastatic at presentation (mCRPC).

**Figure S9. Phylogenetic trees for 25 prostate cancers.** Cluster numbers refer to PyClone clusters. Circle size corresponds to number of mutations present in cluster.

**Supplementary References**

1. Warren AY, Whitaker HC, Haynes B et al. Method for sampling tissue for research which preserves pathological data in radical prostatectomy. Prostate 2013; 73: 194-202.

2. Marafioti T, Paterson JC, Ballabio E et al. Novel markers of normal and neoplastic human plasmacytoid dendritic cells. Blood 2008; 111: 3778-3792.

3. Akarca AU, Shende VH, Ramsay AD et al. BRAF V600E mutation-specific antibody, a sensitive diagnostic marker revealing minimal residual disease in hairy cell leukaemia. Br J Haematol 2013; 162: 848-851.

4. Marafioti T, Jones M, Facchetti F et al. Phenotype and genotype of interfollicular large B cells, a subpopulation of lymphocytes often with dendritic morphology. Blood 2003; 102: 2868-2876.

5. Brieu N, Pauly O, Zimmermann J et al. Slide-specific models for segmentation of differently stained digital histopathology whole slide images. in Proc. SPIE 9784, Medical Imaging 2016: Image Processing.

6. Li H, Durbin R. Fast and accurate short read alignment with Burrows-Wheeler transform. Bioinformatics 2009; 25: 1754-1760.

7. DePristo MA, Banks E, Poplin R et al. A framework for variation discovery and genotyping using next-generation DNA sequencing data. Nat Genet 2011; 43: 491-498.

8. Koboldt DC, Zhang Q, Larson DE et al. VarScan 2: somatic mutation and copy number alteration discovery in cancer by exome sequencing. Genome Res 2012; 22: 568-576.

9. Li H, Handsaker B, Wysoker A et al. The Sequence Alignment/Map format and SAMtools. Bioinformatics 2009; 25: 2078-2079.

10. Wang K, Li M, Hakonarson H. ANNOVAR: functional annotation of genetic variants from high-throughput sequencing data. Nucleic Acids Res 2010; 38: e164.

11. Van Loo P, Nordgard SH, Lingjaerde OC et al. Allele-specific copy number analysis of tumors. Proc Natl Acad Sci U S A 2010; 107: 16910-16915.

12. Murugaesu N, Wilson GA, Birkbak NJ et al. Tracking the genomic evolution of esophageal adenocarcinoma through neoadjuvant chemotherapy. Cancer Discov 2015; 5: 821-831.

13. Dewhurst SM, McGranahan N, Burrell RA et al. Tolerance of whole-genome doubling propagates chromosomal instability and accelerates cancer genome evolution. Cancer Discov 2014; 4: 175-185.

14. Rausch T, Zichner T, Schlattl A et al. DELLY: structural variant discovery by integrated paired-end and split-read analysis. Bioinformatics 2012; 28: i333-i339.

15. Robinson JT, Thorvaldsdottir H, Winckler W et al. Integrative genomics viewer. Nat Biotechnol 2011; 29: 24-26.

16. Szolek A, Schubert B, Mohr C et al. OptiType: precision HLA typing from next-generation sequencing data. Bioinformatics 2014; 30: 3310-3316.

17. Lundegaard C, Lamberth K, Harndahl M et al. NetMHC-3.0: accurate web accessible predictions of human, mouse and monkey MHC class I affinities for peptides of length 8-11. Nucleic Acids Res 2008; 36: W509-512.

18. Roth A, Khattra J, Yap D et al. PyClone: statistical inference of clonal population structure in cancer. Nat Methods 2014; 11: 396-398.

19. McGranahan N, Furness AJ, Rosenthal R et al. Clonal neoantigens elicit T cell immunoreactivity and sensitivity to immune checkpoint blockade. Science 2016; 351: 1463-1469.

20. Andor N, Graham TA, Jansen M et al. Pan-cancer analysis of the extent and consequences of intratumor heterogeneity. Nat Med 2016; 22: 105-113.

21. Robinson D, Van Allen EM, Wu YM et al. Integrative clinical genomics of advanced prostate cancer. Cell 2015; 161: 1215-1228.

22. Rosenthal R, McGranahan N, Herrero J et al. deconstructSigs: delineating mutational processes in single tumors distinguishes DNA repair deficiencies and patterns of carcinoma evolution. Genome Biol 2016; 17: 31.

23. Alexandrov LB, Nik-Zainal S, Wedge DC et al. Signatures of mutational processes in human cancer. Nature 2013; 500: 415-421.

24. Cooper CS, Eeles R, Wedge DC et al. Analysis of the genetic phylogeny of multifocal prostate cancer identifies multiple independent clonal expansions in neoplastic and morphologically normal prostate tissue. Nat Genet 2015.
